# Levodopa modulates semantic fluency and uniqueness in non‐demented patients with progressive supranuclear palsy

**DOI:** 10.1002/brb3.3606

**Published:** 2024-06-30

**Authors:** Jinghong Ma, Guanyu Zhang, Zhenzhen Zhao, Piu Chan, Zheng Ye

**Affiliations:** ^1^ Department of Neurology Xuanwu Hospital of Capital Medical University Beijing China; ^2^ China Institute of Sport Science Beijing China; ^3^ Department of Geriatrics Center The Fourth People's Hospital of Shenyang Shenyang China; ^4^ Department of Neurobiology Neurology and Geriatrics, Xuanwu Hospital of Capital Medical University, Beijing Institute of Geriatrics Beijing China; ^5^ Institute of Neuroscience, Center for Excellence in Brain Science and Intelligence Technology, Chinese Academy of Sciences Shanghai China

**Keywords:** cognitive impairment, levodopa, progressive supranuclear palsy‐Richardson's syndrome, semantic fluency, uniqueness

## Abstract

**Introduction:**

Semantic fluency is the ability to name items from a given category within a limited time, which relies on semantic knowledge, working memory, and executive function. Similar to patients with Parkinson's disease (PD), patients with progressive supranuclear palsy (PSP) scored lower than healthy adults in the well‐established semantic fluency test. However, it is unclear how unique are the produced words. This study examined the relationship between semantic fluency and words’ uniqueness in patients with PSP.

**Methods:**

Twenty‐seven patients with PSP Richardson's syndrome (PSP‐RS), 37 patients with PD, and 41 healthy controls (HC) performed a standard semantic fluency test (animals), and their verbal responses were audio‐recorded. We used the uniqueness to reflect the ability to produce both original and effective work, that is, creativity.

**Results:**

The PSP‐RS group produced fewer correct words and fewer unique words than the PD and HC groups. Moreover, the correlation between fluency and uniqueness was positive in the HC and PD groups but negative in the PSP‐RS group. Importantly, the actual levodopa dose was positively correlated with the fluency but negatively correlated with the uniqueness in PSP‐RS. The PSP‐RS patients who took a greater dose of levodopa tended to produce more correct words but fewer unique words.

**Conclusions:**

These results suggested that levodopa may modulate semantic fluency and uniqueness in the early stages of PSP‐RS.

## 1 INTRODUCTION

Progressive supranuclear palsy (PSP) is a rare neurodegenerative disorder with a prevalence approximately 5.82/100000 according to an epidemiological study from the Japan (Kawashima et al., 2004). It is associated with prominent intracerebral aggregation of 4R‐tau. The Richardson's syndrome (RS) is the most commonly recognized clinical presentation of PSP (PSP‐RS), characterized by postural instability with early falls, akinetic‐rigid syndrome, and vertical supranuclear gaze palsy. The second most common phenotype is PSP parkinsonism predominant (PSP‐P), characterized by tremor, asymmetric onset, and a moderate initial motor response to levodopa (Williams et al., 2005). Neuropsychological evaluation has been extensively used in PSP patients. For example, the combination of frontal assessment battery and single‐photon emission computed tomography was applied for the differentiation of PSP‐RS and PSP‐P. The results showed that frontal assessment battery was more sensitive than single‐photon emission computed tomography (Alster et al., 2021).

PSP may present initially with semantic disorder (Shea et al., 2014). As a well‐established neuropsychological test, the semantic fluency test is widely used in clinical practice (Ardila et al., 2006). In this test, participants are asked to produce as many different examples as possible from a particular semantic category (e.g., animals) within 1 min. Typically, the number of correct words is counted. Patients with PSP or Parkinson's disease (PD) produced fewer correct words than healthy adults in this test (Azuma et al., 1997; Rosser & Hodges, 1994). Semantic disfluency occurs even at the early stages of PSP and PD (Ma et al., 2023; Zhang et al., 2022). Therefore, the semantic fluency test may be a sensitive tool for early detection of disease.

Different approaches have been applied to evaluate the performance of semantic fluency test. Clustering and switching analyses used two key parameters: the mean cluster size, which is defined as the average number of generated words belonging to the same semantic subcategory (e.g., mammal), and total number of semantic switches between subcategories (Troyer et al., 1997). The PSP and PD patients showed a trend toward a smaller cluster size and produced fewer switches than healthy adults in verbal fluency tests (Chowdhury, 2018; Tröster et al., 1998). Our recent studies used the Speechgraph software based on the graph theory and found that the PSP and PD patients generated denser and smaller speech graphs than healthy adults (Ma et al., 2023; Zhang et al., 2022). Moreover, the graph parameters reflected the severity of motor and non‐motor symptoms of patients.

However, the uniqueness of produced words is usually ignored, which reflects the individual creative thinking. As an extraordinary capacity of human, the creativity is the ability to produce both original (unique, uncommon, unusual, novel) and effective (useful, valuable, fit, appropriate) work (Runco & Jaeger, 2012). This widely accepted definition makes the cognitive aspects reflecting creativity experimentally testable. Nikolai and colleagues used the self‐reported Emotional Creativity Inventory questionnaire and found that the PD patients scored lower than healthy adults in preparedness subscale (Nikolai et al., 2022). Ruggiero and colleagues reported that patients with frontotemporal dementia exhibited lower scores than PD patients and healthy adults in the Divergent Thinking Test (Ruggiero et al., 2019).

In this study, we assessed the semantic fluency and words’ uniqueness of PSP‐RS patients at early stages. All participants performed a standard semantic fluency test and their verbal responses were audio‐recorded. We defined a formula for evaluating the uniqueness of correct words to avoid subjectivity. First, we examined group differences in four parameters (fluency, repetitions, incorrect words, and uniqueness). Second, we explored the relationship between fluency and uniqueness in each group. Third, in PSP‐RS, we investigated the effect of levodopa on fluency and uniqueness.

## 2 MATERIALS AND METHODS

This study was approved by the ethics committee of the Xuanwu Hospital according to the Declaration of Helsinki. Each participant signed a written informed consent before participating in this study.

The methods of this study are similar to our previous study (Ma et al., 2023). Our previous study aimed to assess the PSP‐RS patients’ speechgraph, but this study aimed to assess the PSP‐RS patients’ semantic fluency and uniqueness.

### 2.1 Patients and clinical assessments

We included 27 patients with probable PSP‐RS (Movement Disorder Society Clinical Diagnostic Criteria for PSP; Höglinger et al., 2017) at the Xuanwu Hospital between 2022 and 2024. Inclusion criteria were (1) age 45 to 80 years; (2) education ≥5 years; (3) Hoehn and Yahr Stages 1–3; and (4) Mandarin Chinese speaking. Exclusion criteria were (1) possible current depression (Beck Depression Inventory‐II, BDI‐II > 7) or intake of anti‐depressants; (2) possible dementia (Montreal Cognitive Assessment, MoCA < 21/30) or intake of anti‐dementia drugs; (3) alcohol or drug abuse; (4) a history of epilepsy, stroke, or brain injury; and (5) transient ischemic attack or chronic vascular changes.

All patients were parkinsonian and were levodopa unresponsive for motor symptoms. They were assessed on their regular anti‐parkinsonian drugs, including levodopa (*N* = 24), rasagiline (*N* = 4), and selegiline (*N* = 3). The levodopa equivalent daily dose was calculated using the equation of Tomlinson and colleagues (Tomlinson et al., 2010). The severity of non‐motor and motor symptoms was evaluated with the Movement Disorder Society‐sponsored revision of the Unified Parkinson's Disease Rating Scale (MDS‐UPDRS) Part I and III subscales, respectively. Given the association between excessive daytime sleepiness and cognitive deficits (Ohayon & Vecchierini, 2002), we used the Epworth Sleep Scale to measure daytime sleepiness. Table 1 shows demographic and clinical features and neuropsychological measures. We observed no difference between PSP‐RS and PD groups in MoCA (*p *= .486), though there were significant differences among the three groups.

**TABLE 1:** Demographic and clinical features, and neuropsychological measures of patients and healthy controls (means, standard deviations/ranges, and group differences).

Features/measures	PSP‐RS (*N* = 27)	PD (*N* = 35)	Healthy controls (*N* = 40)	Group differences (*p* values)
Male:Female	14:13	18:17	20:20	.968
Age (years)	63.5 (6.7)	62.3 (8.9)	59.9 (6.4)	.146
Education (years)	10.7 (3.5)	12.1 (3.2)	11.5 (1.8)	.167
Motor symptoms				
MDS‐UPDRS III:Motor examination	32.6 (12.0)	24.5 (13.7)	–	.019
Hoehn and Yahr scale	2.4 (0.6)	1.6 (0.5)	–	<.001* PSP‐RS > PD
Disease duration (years)	1.3 (0–11)	2.1 (0–9)	–	.184
Duration of motor symptoms (years)	3.0 (1–12)	3.3 (0–10)	–	.617
Levodopa equivalent daily dose				
Total (mg/day)	314.8 (172.7)	326.3 (282.0)	–	.844
Levodopa (mg/day)	296.3 (172.2)	264.1 (249.9)	–	.551
Non‐motor functions				
MDS‐UPDRS I:Non‐motor experiences of daily living	10.6 (5.6)	8.1 (4.0)	–	.062
Beck depression inventory‐II	3.4 (2.1)	3.1 (2.2)	2.4 (1.7)	.078
Epworth sleep scale	4.5 (3.8)	2.9 (3.1)	3.2 (2.3)	.157
Montreal cognitive assessment	24.0 (2.4)	24.4 (2.1)	27.8 (1.4)	<.001* PSP‐RS≈PD < HC

*Note*: PSP‐RS, progressive supranuclear palsy‐Richardson's syndrome; PD, Parkinson's disease; MDS‐UPDRS, the Movement Disorder Society‐sponsored revision of the Unified Parkinson's Disease Rating Scale; Group differences, *p* values of Kruskal‐Wallis one‐way analysis of variances or one‐way analysis of variances as appropriate; Disease duration or Duration of motor symptoms of less than 6 months counted as 0 years; post hoc two‐sample *t*‐tests, *p *< .004 Bonferroni correction.

*Significant difference (two‐tailed, *p *< .004 Bonferroni correction for thirteen tests).

### 2.2 Two control groups

We recruited two control groups: 35 age‐ and education‐matched patients with idiopathic PD (Movement Disorder Society Clinical Diagnostic Criteria for PD; Postuma et al., 2015) from Xuanwu Hospital and 40 age‐ and education‐matched healthy controls (HC) from local communities.

The inclusion and exclusion criteria of PD were same as PSP‐RS. All patients were assessed on their regular anti‐parkinsonian drugs, including levodopa (*N* = 23), pramipexole (*N* = 11), piribedil (*N* = 5), entacapone (*N* = 4), selegiline (*N* = 3), and amantadine (*N* = 2). They completed the same clinical and neuropsychological measures as PSP‐RS patients.

For the HC group, exclusion criteria were (1) possible current depression; (2) possible dementia or mild cognitive impairment (MoCA < 26/30); (3) alcohol or drug abuse; and (4) a history of significant neurological or psychiatric disorders. They completed the same measures for cognition, mood, and sleep as patients.

### 2.3 Standard analyses

All participants performed a standard semantic fluency test (animals). Participants were asked to produce as many animals as possible within 1 min. We transcribed their verbal responses and defined four parameters: (1) the number of correct words (fluency); (2) uniqueness: the mean value of the uniqueness of each produced correct word (1/N, where N was the number of participants who said this word in all groups); (3) the number of repetitions; and (4) the number of incorrect words: words beyond the category of animal (e.g., lotus).

### 2.4 Statistical analysis

Data were analyzed with IBM SPSS Statistics 20. First, we examined group differences in the four parameters using one‐way analysis of variances (two‐tailed, *p *< .013 Bonferroni correction for four tests). The analysis of variance had a factor group (PSP‐RS, PD, and HC), and covariates age and education. Significant group differences were followed by post hoc two‐sample *t*‐tests.

Second, we examined the relationship between fluency and uniqueness in each group by correlating the number of correct words and uniqueness values (two‐tailed, *p *< .017 Bonferroni correction for three tests).

Third, in the PSP‐RS group, we examined the effect of levodopa by correlating the actual levodopa dose with fluency and uniqueness (two‐tailed, *p *< .025 Bonferroni correction for two tests).

## 3 RESULTS

### 3.1 Group differences in the four parameters

**FIGURE 1:**
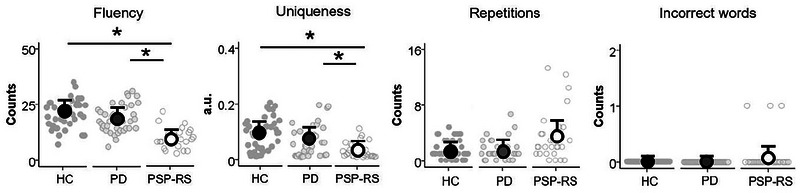
Individual data, group means, and standard errors of fluency, repetitions, incorrect words, and uniqueness in patients with progressive supranuclear palsy‐Richardson's syndrome (PSP‐RS), patients with Parkinson's disease (PD), and healthy controls (HC). *p < .013 Bonferroni correction.

Figure 1 shows the four parameters in each group. Group differences were found in the fluency (*F*[2, 97] = 25.59, *p <* .001, *η_p_* ^2 ^= 0.35), repetitions (*F*[2, 97] = 5.55, *p = *.005, *η_p_* ^2 ^= 0.10), and uniqueness (*F*[2, 97] = 8.65, *p <* .001, *η_p_* ^2 ^= 0.15), but not in the number of incorrect words (*F*[2, 97] = 4.03, *p = *.021, *η_p_* ^2 ^= 0.08). The PSP‐RS group produced fewer correct words (PD: *t*[60] = −6.46, *p <* .001; HC: *t*[65] = −8.42, *p <* .001) and fewer unique words than the PD and HC groups (PD: *t*[60] = −3.21, *p = *.002; HC: *t*[65] = −5.59, *p <* .001). Only the PSP‐RS group produced incorrect words (*N* = 3).

### 3.2 Correlations between the fluency and uniqueness in each group

**FIGURE 2:**
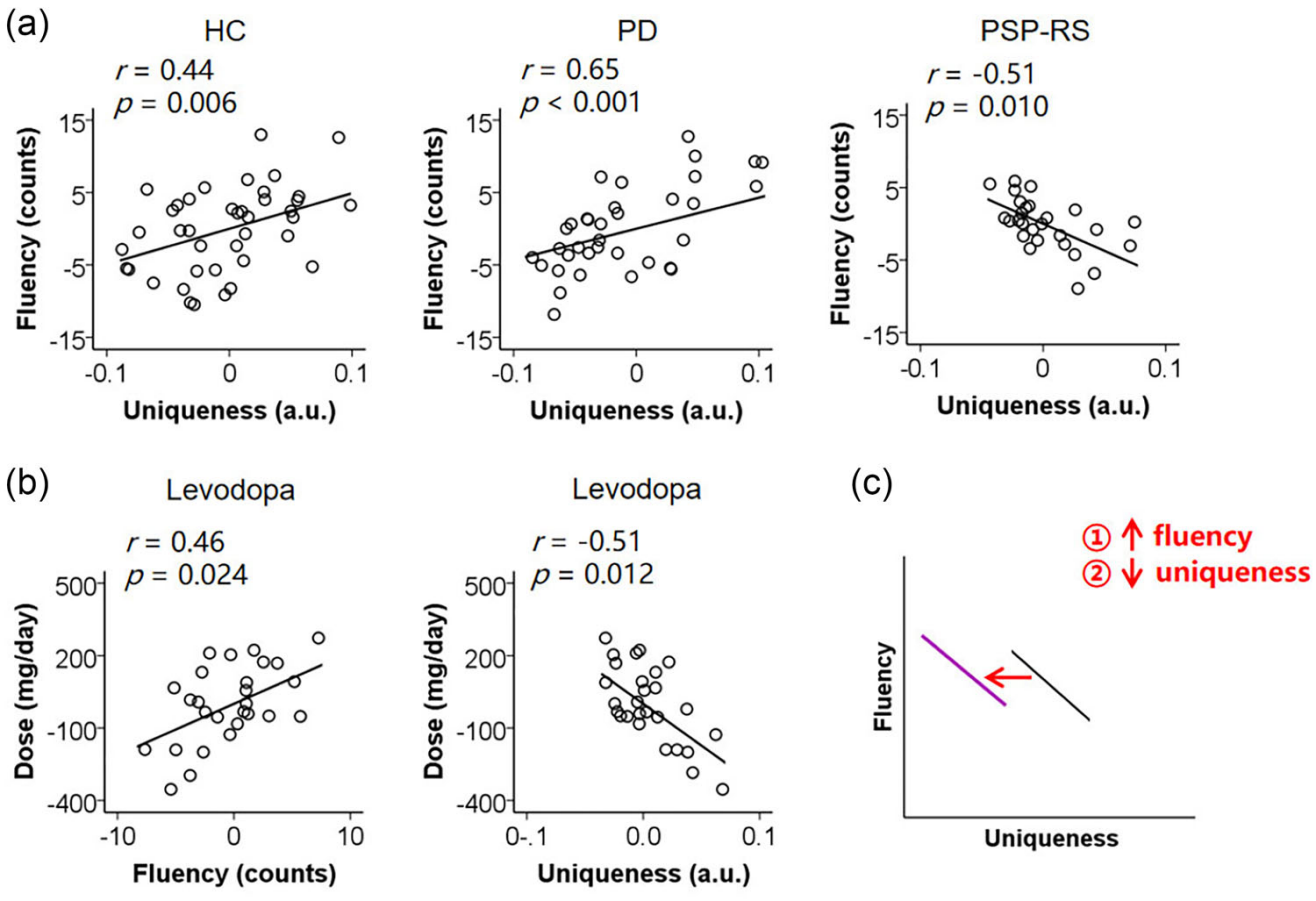
(a) In the healthy controls (HC) and Parkinson's disease (PD) groups, the fluency positively correlated with uniqueness when the age and education were controlled. In the PSP‐RS group, the fluency negatively correlated with uniqueness when the Age and Education were controlled. (b) In the progressive supranuclear palsy‐Richardson's syndrome (PSP‐RS) group, the actual levodopa dose positively correlated with the fluency but negatively correlated with the uniqueness when the levodopa equivalent dose for other drugs, age, and education were controlled. The unstandardized residuals for data were used and values were demeaned. (c) A schematic diagram of effects of levodopa on fluency and uniqueness in PSP‐RS.

Figure 2a shows correlations between the fluency and uniqueness in each group. In the HC and PD groups, we found that the fluency positively correlated with uniqueness when the age and education were controlled (HC: *r *= 0.44, *p *= .006; PD: *r *= 0.65, *p *< .001). In the PSP‐RS group, however, the fluency negatively correlated with uniqueness when the age and education were controlled (*r *= −0.51, *p *= .010). PSP‐RS patients tended to either generate more common words or fewer unique words.

### 3.3 Effects of levodopa on fluency and uniqueness in PSP‐RS

Figure 2b shows the effects of levodopa on fluency and uniqueness in PSP‐RS. We found that the actual levodopa dose positively correlated with fluency (*r *= 0.46, *p *= .024) but negatively correlated with uniqueness (*r *= −0.51, *p *= .012) when the levodopa equivalent dose of other drugs, age, and education were controlled. The PSP‐RS patients who took a greater dose of levodopa tended to produce more correct words but fewer unique words.

## 4 DISCUSSION

In this study, we evaluated semantic fluency and uniqueness in non‐demented patients with early PSP‐RS. We found that PSP‐RS patients produced fewer correct words and fewer unique words than the PD patients and healthy adults in the semantic fluency test. Moreover, in PSP‐RS, the fluency negatively correlated with uniqueness, which was opposite to the PD patients and healthy adults. The PSP‐RS patients tended to either produce more common words or fewer unique words. Importantly, in PSP‐RS, the actual levodopa dose positively correlated with fluency but negatively correlated with uniqueness. Levodopa may help to increase the number of produced words but at the cost of uniqueness in PSP‐RS patients (Figure 2c).

Prefrontal cortex has long been considered as the neural basis of option production (fluency) and the uniqueness of option (Dietrich & Kanso, 2010; Gonen‐Yaacovi et al., 2013). Kaiser and colleagues reported that the anterior prefrontal cortex was involved in generating options for simple real‐world scenarios (Kaiser et al., 2013). Shamay‐Tsoory and colleagues showed that patients with lesions in the medial prefrontal cortex were impaired in originality, that is, the ability to produce infrequent ideas (Shamay‐Tsoory et al., 2011). De Souza and colleagues found that patients with frontal variant of frontotemporal lobar degeneration scored lower than the PD patients and healthy adults in the Torrance Test of Creative Thinking (De Souza et al., 2010). Furthermore, greater creativity was predicted by the white matter integrity of the basal ganglia in healthy adults (Sunavsky & Poppenk, 2020). Compared with the PD patients and healthy adults, the trade‐off between fluency and uniqueness in PSP patients may result from more severe frontal‐basal ganglionic dysfunction (Sachin et al., 2008). An functional magnetic resonance imaging (fMRI) study used force production paradigm analysis and showed that the frontal cortex and basal ganglia were more hypoactive in PSP than in PD patients and healthy adults (Burciu et al., 2015). In addition, PSP patients exhibited profoundly decreased glucose metabolism in the basal ganglia compared with PD patients and healthy adults (Martí‐Andrés et al., 2020).

The temporal lobe also contributes to semantic fluency. A case study reported that the PSP patient with semantic dementia exhibited marked atrophy of the temporal lobe, especially anterior inferior temporal gyrus, and neuronal gliosis/loss was prominent at temporal pole and in inferior and middle temporal gyri (Snowden et al., 2019). Moreover, semantic dementia is usually associated with the pathology consisting of sparse but frequent long, neuritic neuronal cytoplasmic inclusions within temporal cortex. Additionally, striking tau burden in frontal and temporal cortex associates with reduced synaptic density, leading to cognitive impairment (Jellinger, 2023).

Neurochemical mechanisms of semantic fluency and uniqueness are still unclear. A theoretical proposition proposed by Boot et al. (2007) and colleagues indicated that the fronto‐striatal dopaminergic activity might associate with the balance between the flexibility and persistence. To be specific, moderate levels of striatal dopamine facilitate flexible processes (originality, i.e., uniqueness) by the nigrostriatal pathway, while moderate levels of prefrontal dopamine facilitate persistent process (convergent products within a particular category, i.e., fluency) by the mesocortical pathway.

This model was supported by neurobiological studies. Frank proposed that dopamine dynamically modulates the threshold of the response to particular stimuli in basal ganglia, which further influences information updating (Frank, 2005). Vijayraghavan et al. (2007) and colleagues reported that moderate dopamine D1 receptor stimulation in prefrontal cortex could enhance spatial working memory by reducing distractibility. Dual‐state theory (Durstewitz & Seamans, 2008) showed that dopamine D1 receptor, the most prevalent receptor type in the prefrontal cortex (Lidow et al., 1991), favors robust maintenance of information, while the dopamine D2 receptor, the most prevalent receptor type in the striatum (Camps et al., 1989), is beneficial for flexible and fast switching. Brusa and colleagues showed that levodopa, but not dopamine D2 receptor agonist pramipexole, could improve the semantic fluency in PD patients (Brusa et al., 2003). Moreover, another implication of this model is that the fronto‐striatal circuits bias toward flexible processes with the increase in striatal dopamine, while the fronto‐striatal circuits bias toward persistent processes with the increase in prefrontal dopamine (Boot et al., 2017; Cools et al., 2007; Dodds et al., 2008). Inevitably, there were conflicting findings (Cerruti & Schlaug, 2009; Mayseless & Shamay‐Tsoory, 2015). Although our results are inadequate to confirm this model, we suggest that levodopa modulates the semantic fluency and uniqueness in PSP.

This study has limitations. First, the semantic fluency test requires searching for semantic knowledge, which is influenced by individual cultural bias. Although we recruited age‐ and education‐matched control groups, it is difficult to guarantee that the PSP‐RS group's linguistic ability, executive function, and working memory were same as the two control groups. Second, this study lacks sufficient neuropathological data of PSP‐RS patients. It is meaningful to explore the relationship between neuropathological investigation and semantic deficits in PSP. Third, it cannot be denied that the uniqueness approach depends on individual background, exposure, and education, though the 1/N rule is widely applied in mathematical and financial fields. In contrast to optimizing models, this rule produces smaller deviation and performs better in the dataset (Demiguel et al., 2009).

## 5 CONCLUSION

In this study, we used the semantic fluency test to assess the semantic fluency and uniqueness in patients with early PSP‐RS. PSP‐RS patients generated fewer correct and unique words than the PD patients and healthy adults. Moreover, PSP‐RS patients displayed a trade‐off between fluency and uniqueness. Importantly, levodopa may contribute to the balance between producing more words against producing more unique words in PSP‐RS. Future pharmacological studies can explore the effect of antiparkinsonian drugs on PSP patients’ semantic fluency and uniqueness, and corresponding cortical activity.

## AUTHOR CONTRIBUTIONS

**Jinghong Ma**: Investigation; writing—review and editing. **Guanyu Zhang**: Data curation; formal analysis; writing—original draft. **Zhenzhen Zhao**: Investigation; writing—review and editing. **Piu Chan**: Funding acquisition; writing—review and editing. **Zheng Ye**: Conceptualization; funding acquisition; writing—review and editing.

## CONFLICT OF INTEREST STATEMENT

The authors declare no conflicts of interest.

### PEER REVIEW

The peer review history for this article is available at https://publons.com/publon/10.1002/brb3.3606

## Data Availability

The data used to support the findings of this study are included within the manuscript. Further information is available from the corresponding author Guanyu Zhang on request.
